# Piezo1 activates noncanonical EGFR endocytosis and signaling

**DOI:** 10.1126/sciadv.adi1328

**Published:** 2023-09-27

**Authors:** Carlos Pardo-Pastor, Jody Rosenblatt

**Affiliations:** ^1^ Randall Centre for Cell & Molecular Biophysics, New Hunt’s House, School of Basic & Medical Sciences, Faculty of Life Sciences & Medicine, King’s College London, SE1 1UL London, UK.; ^2^ School of Cancer & Pharmaceutical Sciences, Faculty of Life Sciences & Medicine, King’s College London, SE1 1UL London, UK.

## Abstract

EGFR-ERK signaling controls cell cycle progression during development, homeostasis, and disease. While EGF ligand and mechanical inputs can activate EGFR-ERK signaling, the molecules linking mechanical force to this axis have remained mysterious. We previously found that stretch promotes mitosis via the stretch-activated ion channel Piezo1 and ERK signaling. Here, we show that Piezo1 provides the missing link between mechanical signals and EGFR-ERK activation. While both EGF- and Piezo1-dependent activation trigger clathrin-mediated EGFR endocytosis and ERK activation, EGF relies on canonical tyrosine autophosphorylation, whereas Piezo1 involves Src-p38 kinase-dependent serine phosphorylation. In addition, unlike EGF, ex vivo lung slices treated with Piezo1 agonist promoted cell cycle re-entry via nuclear ERK, AP-1 (FOS and JUN), and YAP accumulation, typical of regenerative and malignant signaling. Our results suggest that mechanical activation via Piezo1, Src, and p38 may be more relevant to controlling repair, regeneration, and cancer growth than tyrosine kinase signaling via canonical EGF signaling, suggesting an alternative therapeutic approach.

## Teaser

Mechanical EGFR activation via Piezo1 and Src-p38 triggers neoplastic signaling independent of tyrosine autophosphorylation.

## INTRODUCTION

Mechanical regulation of cell proliferation, differentiation, and migration was originally described decades ago (*1*–*3*), but only recently have molecules that integrate mechanics and signaling come to light. The mechanosensitive Piezo ion channels and Yes-Associated Protein 1 (YAP) transcriptional regulator are the most noteworthy in this integration (*4*–*6*). Human Piezo1 and 2 are mechanically activated cation channels, abundant in a wide variety of tissues and cell types (*4*), that mediate extracellular calcium (Ca^2+^) influx in response to cell confinement (*7*), stretch (*8*), shear stress (*9*, *10*), or matrix stiffness (*11*, *12*). Piezo-dependent Ca^2+^ entry is critical for development, regeneration, and cancer through its roles in cell migration (*7*, *11*, *13*), matrix remodeling (*11*, *14*), cytokinesis (*8*), or stiffness-dependent nuclear translocation of YAP (*11*, *12*). We found that Piezo1 promotes epithelial cell extrusion in response to crowding (*15*) and rapid cell division (G_2_/M transition) in response to stretch (*16*). Moreover, stretch also promotes cell exit from quiescence (G_0_/G_1_ transition) and genome replication (G_1_/S transition) (*17*), both requiring Piezo1 (*18*). These studies strengthen the role of mechanical forces and Piezo1 as master regulators of the cell cycle.

Soluble growth factors regulate cell function through membrane receptors, too. Epidermal growth factor receptor (EGFR) is a widely expressed receptor tyrosine kinase that autophosphorylates its C-terminal cytoplasmic tyrosine residues after binding extracellular soluble ligands (*19*–*24*). These modified residues act as docking sites for adaptor proteins containing phosphotyrosine (pY)–binding Src homology 2 (SH2) domains, such as SH2 domain–containing 1 (SHC) and growth factor receptor–bound protein 2. Both endocytic and effector proteins bind pY-bound adaptors, and their specific combination and phosphorylation by EGFR triggers receptor clathrin-mediated endocytosis (CME) and downstream signaling pathways, such as rat sarcoma/mitogen-activated protein kinase (MAPK), that impact cell migration, cycling (cell division, quiescence, and replication), and malignancy (*19*–*21*, *23*, *25*–*34*). In response to high ligand doses, EGFR becomes down-regulated by a ubiquitin-dependent, nonclathrin endocytic mechanism that targets the receptor for degradation (*24*, *35*, *36*).

Mechanical signals such as cell stretch (*25*, *26*, *32*), apicobasal compression (*37*), matrix-cell adhesion and cell spreading (*28*, *29*, *38*, *39*), or substrate stiffness (*20*, *27*, *31*) can also activate EGFR endocytosis and signaling. However, little is known about molecules that transduce mechanical forces into EGFR signaling and which EGFR pathways mechanical activation induces. As we previously found that Piezo1 controls stretch-activation of the MAPK extracellular signal–regulated kinase 1/2 (ERK1/2) (*16*), a target of EGFR, here we investigate whether Piezo1 provides a molecular link between mechanical and EGFR signaling.

## RESULTS

### Piezo1 triggers CME of EGFR

**Fig. 1.**
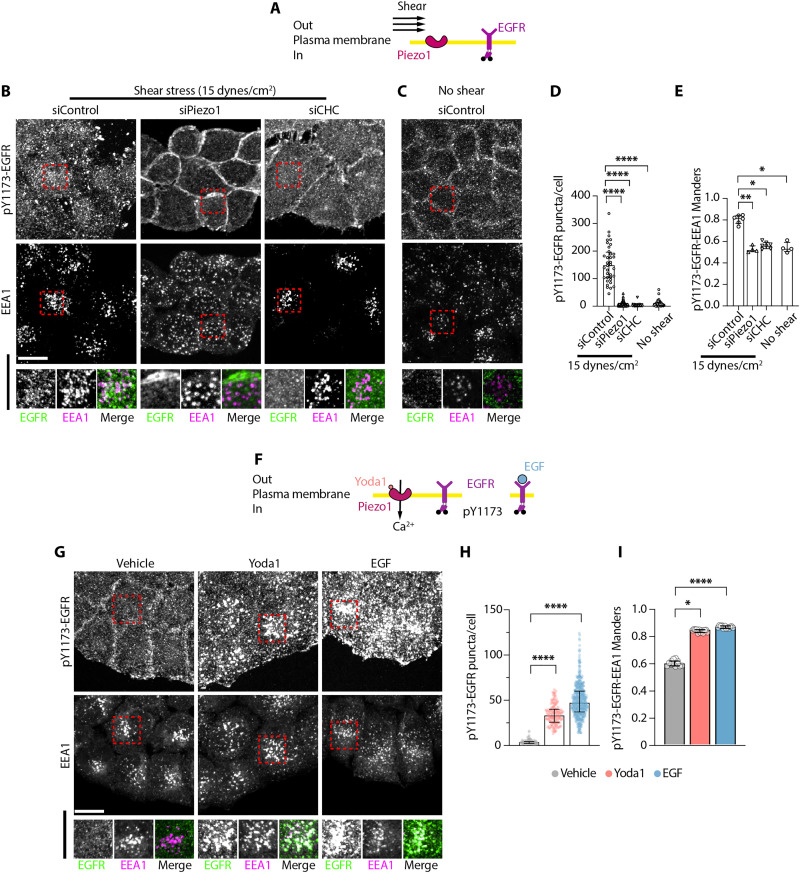
CME of EGFR in response to Piezo1 activation. (**A** and **F**) Schematics of experimental setups. (**B**, **C**, and **G**) Representative maximum intensity projections of HeLa cells immunostained for pY1173-EGFR or EEA1, following the indicated 15-min treatments. (**D** and **H**) pY1173-EGFR puncta per cell, where each symbol represents a cell from *N* ≥ 4 independent experiments [*n* cells for each: (D) siControl, 36; siPiezo1, 161; siCHC, 28; and no shear, 44; (H) siControl: 244 (vehicle), 231 (Yoda1), and 221 (EGF); siPiezo1, 182 (vehicle), 526 (Yoda1), and 192 (EGF); siCHC: 187 (vehicle), 371 (Yoda1), and 234 (EGF)]. (**E** and **I**) Manders colocalization coefficient for pY1173-EGFR and EEA1. Each symbol represents a picture, from *N* ≥ 4 independent experiments with ≥20 cells per picture [*n* pictures for each: (E) siControl, 6; siPiezo1, 4; siCHC, 7; and no shear, 4; (I) 12 for each treatment]. Scale bar, 20 μm. Error bars = median ± interquartile range. ns, nonsignificant, *P < 0.05, **P < 0.01, and ****P < 0.0001 in one-way ANOVA followed by Kreskas-Wallis post hoc test with Dunn’s correction for multiple comparisons.

To investigate the role of Piezo1 in mechanical activation of EGFR signaling, we exposed cells to shear stress, a Piezo1-activating mechanical stimulus (*9*, *10*), and monitored internalization of EGFR phosphorylated on tyrosine-1173 (pY1173-EGFR), an indicator of active EGFR signaling (*20*, *38*, *40*, *41*). We chose HeLa cells because they endogenously express Piezo1 and are widely used for studying EGFR (*24*, *36*, *41*–*43*). Serum-starved HeLa cells exposed to shear stress for 15 min redistributed pY1173-EGFR from the cell periphery to intracellular puncta that colocalize with the early endosome marker early endosome antigen 1 (EEA1) (Fig. 1, A to E). To test whether shear stress–dependent EGFR signaling requires Piezo1, we knocked down Piezo1 with small interfering RNA (siRNA), confirming its loss by the suppression of Ca^2+^ transients in response to the synthetic Piezo1 agonist, Yoda1 (*44*) (fig.S1, A to C). siRNA-mediated knockdown of Piezo1 (siPiezo1) or clathrin heavy chain [siCHC; key endocytic protein (*24*, *36*)], impaired shear stress–induced pY1173-EGFR internalization and colocalization with EEA1, similar to unstimulated siControl cells (Fig. 1, B to E), showing that mechanical activation of EGFR endocytosis requires Piezo1 and clathrin.

To directly activate Piezo1 without triggering other mechanosensitive pathways, we treated serum-starved HeLa cells with the Piezo1 agonist Yoda1. Both 10 μM Yoda1 or EGF (10 ng/ml; as positive control) redistributed pY1173-EGFR from the cell periphery to intracellular EEA1^+^ puncta within 15 min (Fig. 1, F to I). siPiezo1 suppressed responses to Yoda1 but not to EGF, whereas CHC knockdown suppressed pY1173-EGFR internalization and colocalization with EEA1 for both treatments (fig. S1, D to H). Moreover, Yoda1-induced EGFR internalization in human lung adenocarcinoma A549 and canine kidney Madin-Darby canine kidney (MDCK) cells (fig. S2A), widely used in EGFR studies (*33*, *45*–*47*), suggesting a conserved pathway in different cell types and species. A phosphorylation-independent EGFR antibody further confirmed that Piezo1 activation causes EGFR internalization (fig. S2B). Together, these results demonstrate that mechanical stimuli and Piezo1 agonist trigger Piezo1-dependent EGFR internalization into early endosomes via CME, a pathway shared with canonical EGFR signaling in response to low physiological EGF doses (*24*).

### EGFR kinase activity and tyrosine phosphorylation are dispensable for Piezo1-triggered EGFR endocytosis

**Fig. 2.**
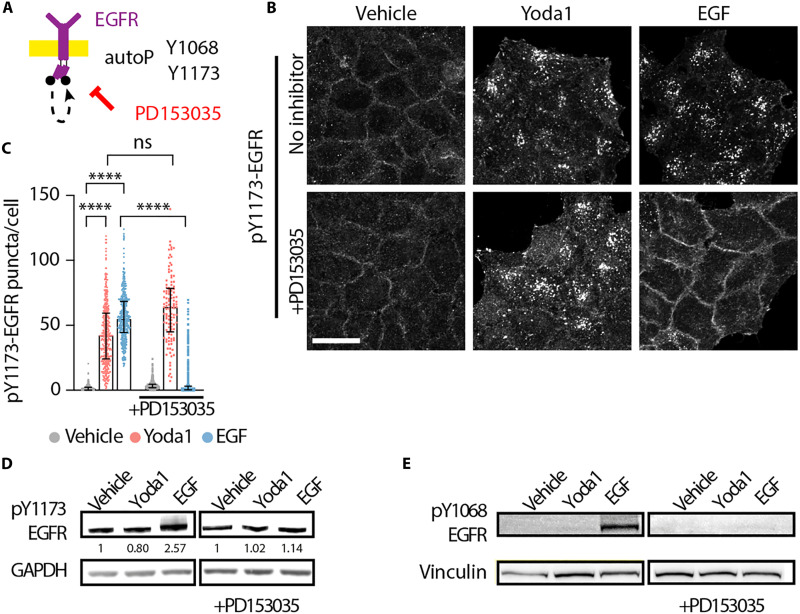
EGFR kinase activity and tyrosine phosphorylation are dispensable for Piezo1-triggered EGFR endocytosis. (**A**) Schematic of the experimental setup. (**B**) Representative maximum intensity projections of pY1173-EGFR stainings in HeLa cells treated for 15 min with vehicle, 10 μM Yoda1, or EGF (10 ng/ml) with or without 30-min preincubation with 1 μM PD153035 (EGFR kinase inhibitor). (**C**) Counts of pY1173-EGFR puncta per cell after the indicated treatments. Each symbol represents a cell, with the following *n*: vehicle, 2725; Yoda1, 860; EGF, 1766; PD, 2136; Yoda1 + PD, 405; EGF + PD, 2410; from six experiments. (**D** and **E**) Representative Western blots of pY1173- and pY1068-EGFR. GAPDH and vinculin used as loading controls. Scale bar, 20 μm. Error bars = median ± interquartile range. ****P < 0.0001 in one-way ANOVA followed by Kruskal-Wallis post hoc test with Dunn’s correction for multiple comparisons.

Both canonical ligand-dependent EGFR phosphorylation and internalization or its transactivation by other transmembrane proteins, Ca^2+^ signaling, or mechanical forces rely on ligand-receptor interaction and EGFR tyrosine autophosphorylation (*22*, *23*, *25*, *37*). Given that Piezo1 is a transmembrane protein that mediates Ca^2+^ influx in response to mechanical forces (*4*), we tested whether Piezo1-dependent EGFR signaling requires receptor autophosphorylation. As expected (*25*, *46*), the EGFR kinase inhibitor PD153035 prevented EGFR internalization and its colocalization with EEA1 in response to EGF. However, it did not affect responses to Yoda1 (Fig. 2, A to C, and fig. S2, C to E). In addition, Yoda1 treatment did not increase pY1068- or pY1173-EGFR, two major autophosphorylation sites (Fig. 2, D and E) (*20*, *38*, *40*, *41*, *46*). Thus, we conclude that EGFR tyrosine kinase activity and canonical tyrosine phosphorylation are dispensable for Piezo1-initiated EGFR endocytosis.

### Piezo1 activates noncanonical EGFR serine phosphorylation by an SFK-p38 kinase axis

**Fig. 3.**
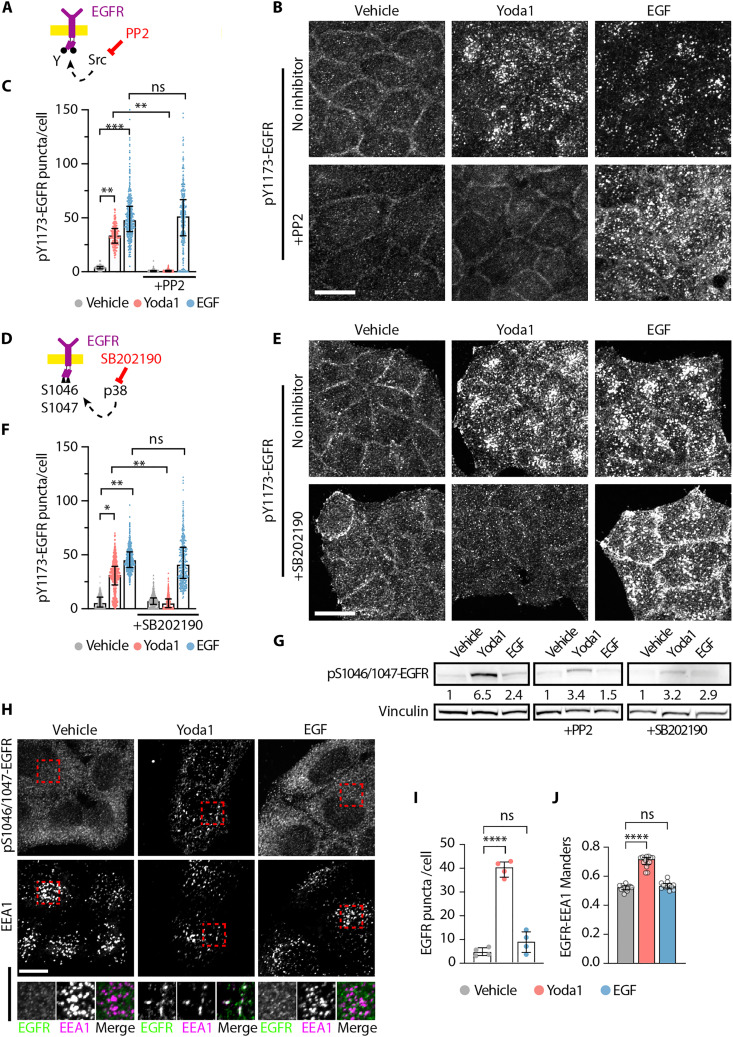
Piezo1 activates noncanonical EGFR serine phosphorylation by an SFK-p38 kinase axis. (**A** and **D**) Schematic of the experimental setups. (**B**, **E**, and **H**) Representative maximum intensity projections of pY1173-EGFR (B and E) or pSer1046/1047-EGFR (H) stainings in HeLa cells treated for 15 min with vehicle, 10 μM Yoda1, or EGF (10 ng/ml) with or without 30-min preincubations with 200 nM PP2 (SFK inhibitor, B), or 10 μM SB202190 (p38 inhibitor, E). (**C** and **F**) Counts of pY1173-EGFR puncta per cell after the indicated treatments. Each symbol represents a cell, with *n*: vehicle, 1143; Yoda1, 717; EGF, 32; PP2, 1694; Yoda1 + PP2, 887; and EGF + PP2, 829; six experiments (C); vehicle, 867; Yoda1, 1125; EGF, 1544; SB, 948; Yoda1 + SB, 1358; and EGF + SB, 1016; three experiments (F). (**G**) Representative Western blots of pS1046/1047-EGFR. Vinculin used as loading control. (**I**) Counts of pSer1046/1047-EGFR puncta per cell after the indicated treatments. Each symbol represents an independent experiment (*N* = 4), with ≥20 cells per picture and *n* = 4 pictures per experiment. (**J**) Manders colocalization coefficient for pSer1046/1047-EGFR and EEA1. Each symbol represents a picture (*n* for each = vehicle, 13; Yoda1, 14; and EGF, 12), from *N* ≥ 4 independent experiments with ≥20 cells per picture. Scale bars, 20 μm. Error bars = median ± interquartile range. *P < 0.05, **P < 0.01, ***P < 0.001, and ****P < 0.0001 one-way ANOVA followed by Kruskal-Wallis post hoc test with Dunn’s correction for multiple comparisons.

Cell-matrix adhesion regulates EGFR via the Src family kinases (SFK) (*29*, *38*) and can bypass EGFR kinase activity (*39*), suggesting an alternative signaling axis to regulate EGFR in response to Piezo1 activation. HeLa cell pretreatment with the SFK inhibitor PP2 suppressed Yoda1-induced pY1173-EGFR internalization and colocalization with EEA1 but did not affect responses to EGF (Fig. 3, A to C, and fig. S3, A, B, and D). This shows that EGFR endocytosis in response to Yoda1 requires SFK activity, unlike EGF-mediated signaling. Although adhesion-dependent EGFR signaling requires SFK-dependent phosphorylation of EGFR on Y1068 (*39*), we find that Yoda1 does not alter pY1068-EGFR levels (Fig. 2E). Therefore, we investigated other noncanonical mechanisms that might control EGFR endocytosis by Piezo1.

Cellular stresses can activate p38 MAPK to phosphorylate EGFR serine residues 1046 and 1047 (pS1046/1047-EGFR) and recruit the endocytic machinery [e.g., activator protein 2 (AP-2), clathrin] to internalize EGFR (*34*, *42*, *45*, *46*, *48*–*50*). Because Piezo1 can activate p38 (*51*), we tested whether Piezo1-dependent EGFR endocytosis requires p38. We find that inhibiting p38 with SB202190 suppresses EGFR endocytosis in response to Yoda1 but not to EGF (Fig. 3, D and F, and fig. S3, A, C, and D). In addition, immunoblots indicate that Yoda1 but not EGF increases pS1046/1047-EGFR in an SFK- and p38-dependent fashion (Fig. 3G). Accordingly, Yoda1 but not EGF also increased the number of pS1046/1047-EGFR^+^ early endosomes (Fig. 3, H to J). Thus, Piezo1 activation leads to SFK- and p38-dependent EGFR serine phosphorylation to drive receptor endocytosis, a pathway shared with cell stress (*34*, *42*, *45*, *46*, *48*–*50*).

### Functional EGFR internalization in response to Piezo1 activation leads to different nuclear signals

**Fig. 4.**
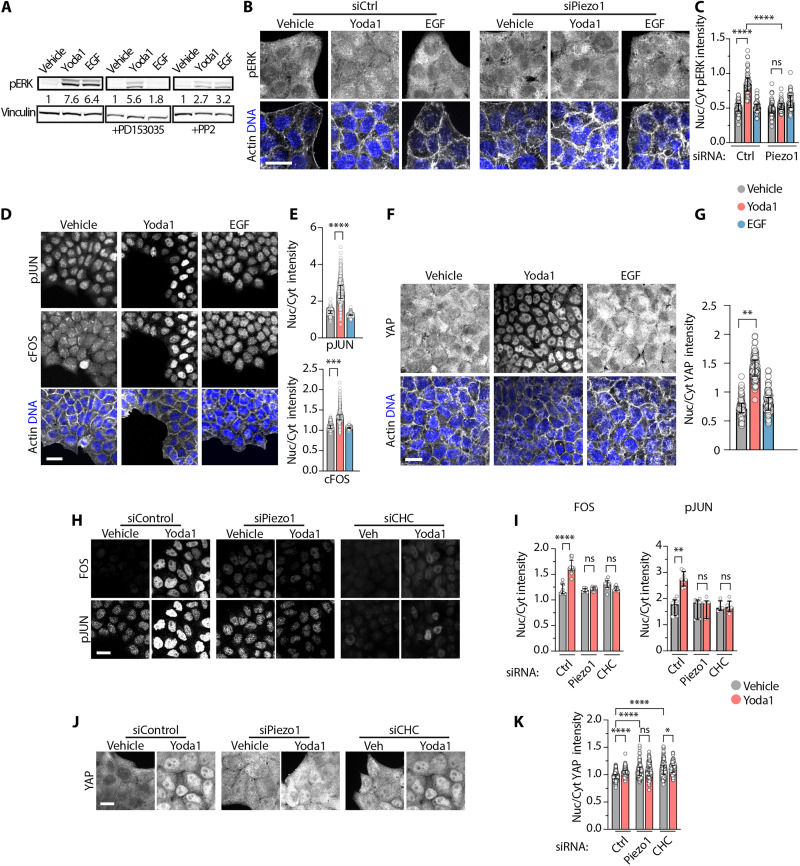
Functional EGFR internalization in response to Piezo1 activation leads to different nuclear signals. (**A** and **B**) Representative western blot (A) and immunostainings (B) of pERK1/2 after 15 min of the indicated treatments, with vinculin as a loading control in (A) and DNA and actin serve as spatial references in (B). (**C**) Quantification of the nuclear versus cytoplasmic (Nuc/Cyt) enrichment of pERK1/2 signal. (**D**, **F**, **H**, and **J**) Representative immunostainings of pJUN and FOS (D and H) or YAP (F and J) after 2 hours of the indicated treatments. DNA and actin shown as spatial references. (**E** and **G**) Quantification of the nuclear versus cytoplasmic (Nuc/Cyt) enrichment of FOS and pJUN (E) or YAP (G) signal. Each symbol in (C), (E), (G) and (**K**) represents a cell from ≥3 independent experiments, with the following *n*s: vehicle: 91 (C, siCtrl), 146 (C, siPiezo1), 3538 (E), 177 (G), 420 (K, siCtrl), 113 (K, siPiezo1), and 205 (K, siCHC); Yoda1: 152 (C, siCtrl), 134 (C, siPiezo1), 2543 (E), 301 (G), 420 (K, siCtrl), 113 (K, siPiezo1), and 193 (K, siCHC); EGF: 160 (C, siCtrl), 139 (C, siPiezo1), 3110 (E), and 283 (G). In (**I**), each symbol represents a picture, with ≥200 cells per picture, from *N* ≥ 3 independent experiments, with the following *n*s: vehicle: 9 (siC, FOS), 8 (siPiezo1 and FOS), 8 (siCHC and FOS), 8 (siC and JUN), 7 (siPiezo1 and JUN), and 4 (siCHC and JUN); Yoda1: 12 (siC and FOS), 12 (siPiezo1 and FOS), 9 (siCHC and FOS), 8 (siCtrl and JUN), 6 (siPiezo1 and JUN), and 5 (siCHC and JUN). Scale bars, 20 μm. Error bars = median ± interquartile range. *P < 0.05, **P < 0.01, ***P < 0.001, and ****P < 0.0001 in one-way ANOVA followed by Kruskal-Wallis post hoc test with Dunn’s correction for multiple comparisons.

ERK1/2 MAPK activation is a hallmark of EGFR signaling, with amplitude, duration, and location determining ERK1/2 signaling outcomes (*30*, *52*). To investigate how Piezo1 activation of EGFR signaling affects ERK1/2 signaling, we next followed total ERK1/2 activation and localization. Immunoblots indicate that both Yoda1 and EGF activation of EGFR promote similar levels of (active) phosphorylated ERK1/2 (pERK1/2) (Fig. 4A, left lanes). While the EGFR kinase inhibitor PD153035 suppressed EGF-dependent pERK1/2, it only partially inhibited phosphorylation by Yoda1 (Fig. 4A, center lanes). However, the SFK inhibitor PP2 dampened both Yoda1- and EGF-induced pERK1/2 (Fig. 4A, right lanes). pERK1/2 accumulates within nuclei in response to Yoda1 but not EGF treatment (Fig. 4, B and C). As in previous experiments, siPiezo1 cells did not respond to Yoda1 (Fig. 4, B and C), confirming that Yoda1 relies on Piezo1. Therefore, despite triggering similar pERK1/2 levels, Piezo1- and EGF-dependent EGFR internalization led to different signaling outcomes.

Spatiotemporal dysregulation underlies many steps of tumorigenesis (*19*, *30*). The duration and strength of nuclear pERK1/2 signaling regulates downstream gene expression dynamics, which ultimately drives cell proliferation, differentiation, transformation, and metastasis (*19*, *30*). We found that Piezo1 activation by Yoda1 leads within 2 hours to nuclear accumulation of the dimeric transcription factor AP-1, formed by the products of proto-oncogenes *FOS* and *JUN*, within 2 hours, whereas EGF does not (Fig. 4, D and E). In turn, AP-1 cooperates with YAP, which transcriptionally activates dedifferentiation, regeneration, and malignancy, in response to mechanical signals (*5*, *6*, *53*–*55*) and Piezo1 channel activation (*11*, *12*). Yoda1, but not EGF treatment, caused nuclear YAP accumulation (Fig. 4, F and G). These results show that Piezo1 activation triggers sustained nuclear accumulation of the dimeric transcription factor AP-1 and their mechanosensitive coactivator YAP.

While Piezo1 knockdown prevented all Yoda1-dependent nuclear responses (Fig. 4, H to K), CHC knockdown suppressed only Yoda1-induced nuclear accumulation of AP-1 (Fig. 4, H and I) but not YAP (Fig. 4, J and K). Basal nuclear YAP levels were higher in siPiezo1 or siCHC than in siControl cells (Fig. 4K), suggesting that basal YAP nuclear exclusion requires Piezo1 and CHC, similar to a recent finding (*56*).

**Fig. 5.**
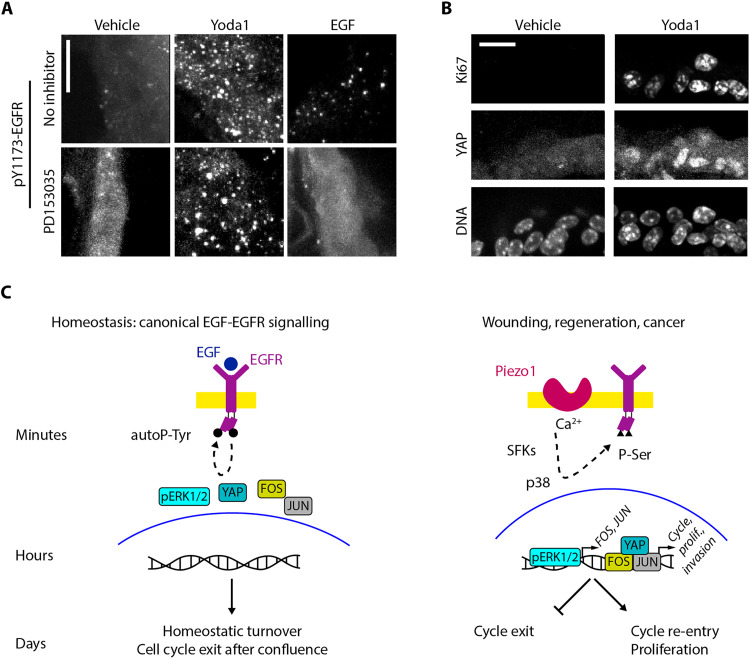
Piezo1 activates noncanonical EGFR endocytosis and YAP signaling ex vivo. (**A** and **B**) Representative mouse lung slices immunostained for pY1173-EGFR after 15 min or for YAP and Ki67 after 48 hours of the indicated treatments. Scale bars, 20 μm. (**C**) Model schematic.

We next investigated whether Piezo1-dependent activation of EGFR and YAP also occurs in tissues using ex vivo mouse lung slices, previously used to study EGFR activation by mechanical stimuli (*37*). While pY1173-EGFR levels were negligible in control samples, 15-min treatments with Yoda1 or EGF increased the number of intracellular pY1173-EGFR puncta in airway cells. Inhibition of the EGFR kinase function with PD153035 suppressed responses to EGF, but not to Yoda1 (Fig. 5A), confirming our cell culture data (Fig. 2). In addition, Yoda1 treatments for 24, 48, or 72 hours increased the number of airway cells positive for both nuclear YAP and the cycling cell marker Ki67, both of which were negligible in control, untreated samples (Fig. 5B). Our lung slice experiments confirm that activating EGFR via EGF versus Piezo1 trigger vastly different outcomes in epithelial tissue and cultured cells.

## DISCUSSION

We show that Piezo1 activates EGFR through an alternative pathway to the canonical EGF-activation (Fig. 5C). During steady-state cell turnover, EGF activates EGFR signaling via tyrosine autophosphorylation, receptor internalization, and cytoplasmic ERK activation. Here, we find that acute Piezo1-dependent EGFR activation signals SFK/p38/nuclear ERK to transcribe early intermediate genes (*FOS* and *JUN*) and YAP that promote cell cycle entry. In this way, the same receptor can interpret different inputs to relay separate outcomes, depending on the function of its tyrosine kinase domain. We confirmed these findings in an ex vivo mouse lung slice model over several days.

The regulation we found describes a mechanism consistent with paradoxical signaling, where a given signal can trigger opposing outcomes (*57*). In previous studies, we identified a paradoxical signaling response involving Piezo1, depending on its location and the force it experiences: In stretched cells, Piezo1 is mainly found at the plasma and nuclear membranes and promotes cell division, whereas in crowded cells, the channel forms uncharacterized intracellular structures that promote cell extrusion and death (*15*, *16*). Similarly, EGFR location determines signaling dynamics: At the plasma membrane, EGFR promotes cell migration, whereas at endosomes, it stimulates proliferation (*52*, *58*–*61*). Moreover, AP-1 induction requires EGFR at endosomes, without its kinase activity or Tyr phosphorylation (*40*). Our inhibitor and siRNA experiments show that Piezo1-dependent EGFR signaling (e.g., ERK activation and AP-1 induction) requires EGFR internalization via CME but not its kinase activity or Y1173 autophosphorylation (Figs. 2 and 4). This favors a model where EGFR endocytosis is key for AP-1 induction in response to Piezo1 activation. Further experiments knocking down endosome constituents and EGFR are needed to clarify these aspects.

Our inhibitor data identify SFK and p38 as key intermediates for Piezo1 activation of EGFR signaling. Our findings support previous studies showing roles for p38 and EGFR in prosurvival and regenerative signaling, rather than known ligand-induced canonical EGFR responses (*45*, *46*, *48*, *49*, *62*–*64*). Previous work identified several exogenous stressors (antibiotics, anticancer drugs, ultraviolet radiation, and *Candida* infection) as activators of this SFK-p38-EGFR axis; however, our work proposes endogenous mechanical forces activating this same axis via Piezo1.

Nevertheless, the mechanisms linking Piezo1 activation to SFK and p38 remain unclear. The SFK member Fyn, the Ca^2+^-dependent protease chaplain, and Ca^2+^/calmodulin-dependent kinase II are relevant candidates because all three are activated by Piezo-dependent Ca^2+^ signals and invoked in p38 activation or EGFR signaling in different scenarios (*11*, *65*–*69*). Given the ubiquitous expression of all these molecules and their participation in both cell homeostasis and disease development, several research fields will benefit from further characterization of the Piezo1-SFK-p38 axis using both pharmacological and genetic approaches.

Our findings may be relevant for cancer resistance to anti-EGFR therapy. First, approved EGFR-neutralizing antibodies or inhibitors target the receptor’s kinase activity. However, transformation and bad prognosis are associated with increased mechanical signaling and expression of Piezo1, SFKs, p38, and EGFR but not to EGFR phosphorylation status (*11*, *14*, *70*–*72*). Second, internalized EGFR has prosurvival functions in cancer cells that are kinase-independent and, thus, unaltered by current tyrosine kinase targeting therapies (*46*, *47*, *62*, *72*, *73*). One possibility is that the increased mechanical forces cancer cells experience as they invade and migrate (*7*, *11*, *74*, *75*) will activate the Piezo1-SFK-p38-EGFR axis, thereby promoting cell proliferation and chemo-resistance. In addition, Piezo1 might be promoting endocytosis by its membrane-bending activity (*76*) or by triggering actin polymerization, essential for ensuring endocytosis under high membrane tension (*7*, *11*, *77*, *78*). Manipulation of endocytic pathways for disease treatment is a promising research area and targeting this axis could address an unmet medical need against kinase inhibitor–resistant tumors, e.g., ~50% of non–small cell lung cancers or ~80% of advanced colorectal cancers (*72*).

Our identification of Piezo1 as an initiator of EGFR endocytosis contributes to a growing list of membrane-based processes controlled by this channel that includes endosomal trafficking to the midbody during mitotic abscission (*8*) and phagocytosis (*79*). Membrane tension is both the fundamental activator of Piezo channels (*80*, *81*) and a key determinant of membrane remodeling and trafficking controlling pluripotency, differentiation, homeostatic turnover, and aging (*82*–*84*). Our work adds EGFR to the picture, highlighting the relevance of kinase-independent EGFR-driven mechanisms previously disregarded by a focus on EGFR mutations. All these recent works point to the mechanical control of membrane trafficking as a promising research field and highlight Piezo channels as key players in it.

Our findings may also yield practical laboratory uses. Given that artificial YAP activation can de-differentiate cells into lineage-restricted stem cells and increase organ size (*53*, *85*), our findings that Yoda1 activates YAP signaling and cell cycle entry ex vivo (Fig. 5) suggest a simple method to obtain tissue-specific stem cells in culture or promote tissue growth bypassing current needs to transduce cells with lentiviral vectors (*53*).

While we have identified an important role for mechanical activation of EGFR via Piezo1, one caveat of our work is that the mechanical (shear stress) and chemical (Yoda1 and EGF) stimuli we used may not capture the physical forces that cells ordinarily experience. For instance, cells squeezing through narrow passages may experience both compression and stretch simultaneously and spatiotemporal fluctuations could also affect the outcomes of these signals (*7*, *13*). In addition, EGFR internalization assayed by immunostaining does not reflect changes to its structure or binding partners, both of which determine downstream signaling (ERK activity and FOS transcription) and cell function (differentiation and proliferation) (*19*, *86*).

Our work identifies Piezo1 as a missing link for mechanically activating EGFR and indicates that this activation adopts a different pathway with different outcomes. Mechanical activation of EGFR requires SFK/p38 kinases and serine phosphorylation, rather than EGFR tyrosine phosphorylation, enzymatically separating these two differential outcomes. Given the roles of mechanics in regeneration, oncogenesis, and the increased prevalence of tyrosine kinase inhibitor chemoresistance, our discovery may reveal insight into EGFR signaling during repair and malignancy.

## MATERIALS AND METHODS

Unless noted, reagents were obtained from Thermo Fisher Scientific.

### Cell culture

Nonverified HeLa, A549, and MDCK-II cells were grown in Dulbecco’s modified Eagle’s medium (DMEM, 31966021) with 10% fetal bovine serum (FBS; 10270106) and 1% penicillin/streptomycin (Pen/Strep; 15070063) in a cell culture incubator at 37°C with 5% CO_2_. For all experiments, cells were seeded in growth medium. After 24 hours, growth medium was washed twice with phosphate-buffered saline (PBS; 14190250) and replaced by starvation medium (DMEM + 1% Pen/Strep, FBS omitted) for 24 hours before cell treatment. All cells tested negative for mycoplasma contamination in periodic tests (Sartorius, 20-700-20).

### siRNA transfection

The day after seeding, 20 to 40% confluent cells were transfected with Lipofectamine RNAiMax (13778) and reduced-serum minimal essential medium (OptiMEM, 31985062) following manufacturer’s instructions. siRNA pool (Horizon Discovery: siControl, D-001810-10-50; siPIEZO1, L-020870-03-0050; and siCHC, L-004001-01-0020) final concentration was 10 nM. siRNA transfection was repeated 24 hours later. The next day, cells were trypsinized and seeded on glass coverslips in growth medium before starvation overnight and treatment. Experiments were performed 72 hours after the first siRNA transfection.

### Chemicals and treatments

After overnight serum starvation, cells were treated with dimethyl sulfoxide (DMSO; 276855), 10 μM Yoda1 (Tocris, 5586, reconstituted in DMSO) or EGF (10 ng/ml; PeproTech, 400-25, reconstituted in deionized water) for indicated durations. Experiments involving inhibitors included a 30-min pretreatment with EGFR kinase inhibitor 1 μM PD153035 (Sigma-Aldrich, SML0564), SFK inhibitor 200 nM PP2 (Sigma-Aldrich, P0042), or p38 inhibitor 10 μM SB202190 (Abcam, ab120638), all reconstituted in DMSO. All treatments were prepared reusing starvation medium.

Shear stress was delivered to cells seeded on microfluidic chambers (Ibid 80166, I Luer 0.2 mm height, polymer coating) using a peristaltic pump (Thermo Fisher Scientific, 16609762) with a starvation medium flow rate of 4 ml/min. According to the manufacturer’s instructions, shear stress (τ) is the product of flow rate (ϕ, 4 ml/min), medium’s dynamical viscosity [η ≈ 0.0073 dynes·s/cm^2^ for serum-free DMEM (*87*)] and a chamber-dependent correction factor (512.9), yielding ≈ 15 dynes/cm^2^, a shear stress value shown to activate Piezo1 (*9*, *10*).

### Calcium imaging

Cells grown on glass coverslips were loaded with 4.5 μM of the Ca^2+^-sensitive dye Calbryte 520 AM (AAT Bioquest, 20651, reconstituted in DMSO) for 30 min in the cell culture incubator (37°C, 5% CO_2_), followed by washing and 30 min of additional incubation for AM cleavage and dye equilibration. Coverslips were then mounted on a recording chamber (Warner Instruments, 642420) and intracellular Ca^2+^ levels were imaged at room temperature (RT) using a 20× air objective, 2 × 2 binning, and green fluorescent protein–compatible epifluorescence settings. All solutions were prepared in Fluobrite DMEM (A1896701) supplemented with 20 mM Hepes (15630080). For solution exchange, we used a peristaltic pump connected to the recording chamber.

### Western blot

Cells were seeded on 10-cm culture plates and starved and treated when 60 to 70% confluent. After treatment, plates were placed on ice, washed twice with ice-cold PBS, and lysed in 250 μl per plate of radioimmunoprecipitation assay (RIPA; 89901) buffer supplemented with EDTA and protease (78430) and phosphatase (Merck, 524625) inhibitor cocktails. After 1 min of incubation on ice, plates were scraped and the lysate placed in tubes, followed by 30-min incubation in ice, vortexing every 5 min. Lysates were then centrifuged at 13000 rpm for 10 min at 4°C. The resulting supernatants were placed in new tubes and their protein content quantified with a bicinchoninic acid kit (23225). Fifty micrograms of protein adjusted to 10 μl in RIPA buffer and dying (B0007) buffers was denatured at 95°C for 10 min, spun down, supplemented with sample reducing buffer (B0004), and loaded in 4 to 12% bis-tris gels (NW04120BOX). A well with 5 μl of a prestained protein standard (LC5925) was used to track protein separation during electrophoresis at 120 V for 1.5 hours in MOPS-SDS buffer (B0001). Proteins were then transferred to nitrocellulose membranes (IB23002) with a dry transfer device (IB21001) at 25 V for 8 min. Membranes were blocked for 1 hour at RT with 5% bovine serum albumin (BSA; for phosphorylated targets) or with 5% nonfat powder milk. After overnight primary antibody incubation and three 5-min washings, membranes were incubated at RT for 1 hour with secondary antibodies followed by three additional 5-min washings, 1-min incubation with enhanced chemiluminiscence substrate (32209), and imaging. Band density was later quantified with the Analyze/Gels tool in Fiji (*88*). In some cases, membranes were then stripped and reblotted following the manufacturer’s instructions (Sigma-Aldrich, 2500). A 0.1% Tween 20 (Sigma-Aldrich, P137) in tris(hydroxymethyl)aminomethane-buffered saline (TTBS) was used for blocking solutions and washings.

### Antibodies–Western blot

Primary antibodies are as follows: pY1173-EGFR (R&D Systems, AF1095; RRID: AB_416526), pY1068-EGFR (Cell Signaling Technology, 3777S; RRID: AB_2096270), pS1046/S1047-EGFR (Abcam, ab76300; RRID: AB_1523528), and pERK1/2 (Cell Signaling Technology, 4370S; RRID: AB_2315112) all 1/1000 in 5% BSA-TTBS; and vinculin (700062; RRID: AB_2532280) and glyceraldehyde-3-phosphate dehydrogenase (GAPDH; Abcam, ab8245; RRID: AB_2107448), 1/2000 in 5% fat-free powder milk-TTBS. Incubated overnight at 4°C inside Falcon tubes with constant rotation.

Secondary antibodies are as follows: horseradish peroxidase–conjugated secondary anti-rabbit antibody (65-6120; RRID: AB_88384) diluted 1:2500 in 5% nonfat powder milk-TTBS and incubated 1 hour at RT.

### Lung slice obtention, treatment, and staining

Animal use for this study was approved by the Ethical Review Committee at King’s College London and the Home Office, United Kingdom, according to Animals (Scientific Procedures) Act 1986 [Home Office Project Licensee Training (PPL) number P68983265]. Mouse lung slices were obtained adapting an existing protocol (*89*). C57BL/6J mice (The Jackson Laboratory, strain 000664; RRID: IMSR_JAX:000664) were killed by inhalation of CO_2_ followed by cervical dislocation. After opening the chest cavity, a 20 gauge × 1.25 in canula was inserted in the trachea through a small incision and used to inflate the lungs with 2% low-melting agarose [Thermo Fisher Scientific, BP1360, 2% in Hanks’ balanced salt solution (14025)]. After excision and washing in PBS, the lobes were separated and individually embedded in 4% low-melting agarose. After casting on ice, a Leica VT1200S vibratome was used to cut 200-μm-thick slices. Slices were washed and incubated overnight in DMEM/F-12 (11320033) with 10% FBS and 1% Pen/Strep in the cell culture incubator. The next morning, slices were individually transferred to a 24-well plate and treated with DMSO or 10 μM Yoda1. After the indicated times, treatments were aspirated, and slices washed thrice with PBS and fixed with 4% paraformaldehyde (PFA; 28908) overnight at 4°C. After two 30-min washings, slices were blocked in 0.1% Triton X-100 and 1% BSA. Primary and secondary antibodies (1/100) were consecutively incubated overnight at 4°C before incubation with 1/1000 4′,6-diamidino-2-phenylindole for 20 min and mounting in Prolong Gold (P36930). A 0.5% Triton X-100 was used for three 30-min washings between incubations. All solutions were prepared in PBS.

### Fixed cell staining

After treatment, cells grown on glass coverslips were fixed in 4% PFA for 20 min at 37°C, permeabilized with 0.5% Triton X-100 (Sigma-Aldrich, X-100) for 5 min at RT, and stained with primary (overnight, 4°C) and secondary (45 min, RT) antibodies. Coverslips were mounted with Fluoromount-G (004958-02). All solutions were prepared and washed with PBS.

### Antibodies–staining

Primary antibodies are as follows: pY1173-EGFR (1/250, R&D Systems, AF1095; RRID: AB_416526), pS1046/1047-EGFR (1/250, Abcam, ab76300; RRID: AB_1523528), total EGFR (1/200, antibodies.com, A86603; RRID: AB_2753073), EEA1 (1/250, BD Biosciences, 610457; RRID: AB_397830), phospho-ERK (1/200, Cell Signaling Technology, 4370S; RRID: AB_2315112), YAP (1/200, Santa Cruz Biotechnology, sc-101199; RRID: AB_1131430), phospho-cJUN (1/200, Abcam, ab32385; RRID: AB_726900), cFOS (1/200, Santa Cruz Biotechnology, sc-166940; RRID: AB_10609634), and Ki67 (1/100, Abcam, ab16667; RRID: AB_302459).

Secondary antibodies are as follows: 1/250 Alexa Fluor (AF)–conjugated 488 anti-rabbit (A11008; RRID: AB_143165) and 594 anti-mouse (A11005; RRID: AB_141372), supplemented with 1/500 AF647 phalloidin (A22287) and Hoechst (30 μg/ml; 62249).

For mouse lung slices, 1/100 antibody dilutions were used. All mixes were prepared in 1% BSA (A7906-100G) in PBS.

### Imaging

Samples were imaged at 1-μm-thick Z displacements through 20× and 40× air and 60× oil objectives of a Nikon Eclipse Ti2-E microscope with a Yokogawa CSU-W1 spinning disk system coupled to an Andor DU-888 camera, and a Toptica multilaser bed. Settings remained unchanged between conditions.

### Quantification of pY1173-EGFR internalization

Maximum Intensity Projections (MIP) were built from raw ND2 files using the Extract Images and Make projections functions of the Process images macro set developed by C. Leterrier in Fiji, available at https://github.com/cleterrier/Process_Images. Next, Cell Profiler (*90*) was used to sequentially (i) segment nuclei using the DNA channel, (ii) segment cells on the actin channel using nuclei as seeds, (iii) generate masks of the cytoplasm after shrinking segmented cells to omit cell boundaries, (iv) enhance speckles in the pY1173-EGFR image using a feature size of 10, (v) segment speckles for counting, and (vi) export data as comma-separated values (CSV).

### Quantification of pS1046/1047-EGFR internalization

pS1046/1047-EGFR puncta were manually counted in Fiji from two-color MIP of EEA1 and pS1046/1047-EGFR, using EEA1 as spatial reference for endosomes.

### Colocalization analysis

We opted for an approach generally used for colocalization studies of endosomal proteins, otherwise susceptible to statistical artifacts (*91*). First, we applied a 32 × 32 px median filter to MIPs of EEA1 and pY1173-EGFR (Figs. 1 and 2) or pS1046/1047-EGFR (Fig. 3). The resulting image was subtracted from the original MIPs for background correction. Then, EGFR-EEA1 colocalization was quantified as the Manders overlap coefficient for pY1173-EGFR and EEA1 using the Coloc2 plugin in Fiji.

### Nuclear translocation of YAP, cFOS, and pJUN

Background, intra-, and juxta- nuclear regions of interest (ROIs) were manually drawn with Fiji using the mid-height image of each Z-stack. Median intensity values of each ROI were exported to a CSV file later used for background subtraction and nuclear/cytoplasmic ratio calculation in RStudio (Posit) or Excel (Microsoft).

### Statistical analysis

All graphs and statistical analyses were done with Prism 9.5.1 (GraphPad Software). Data are presented as median ± interquartile range. Given that data distribution was not Gaussian, the statistical significance of the differences between treatments was assessed with one-way analysis of variance (ANOVA) followed by Kruskal-Wallis post hoc test with Dunn’s correction for multiple comparisons, as suggested by the software. The threshold for statistical significance was *P* < 0.05.

## Supplementary Materials

### This PDF file includes:

Figs. S1 to S3 Legend for table S1 Legend for example cell profiler pipeline for EGFR puncta detection

### Other Supplementary Material for this manuscript includes the following:

Table S1

Example cell profiler pipeline for EGFR puncta detection

## References

[1] M. Abercrombie, J. E. Heaysman, Observations on the social behaviour of cells in tissue culture. Exp. Cell Res. 5, 111–131 (1953). 13083622 10.1016/0014-4827(53)90098-6

[2] J. Folkman, A. Moscona, Role of cell shape in growth control. Nature 273, 345–349 (1978). 661946 10.1038/273345a0

[3] F. M. Watt, P. W. Jordan, C. H. O’Neill, Cell shape controls terminal differentiation of human epidermal keratinocytes. Proc. Natl. Acad. Sci. U.S.A. 85, 5576–5580 (1988). 2456572 10.1073/pnas.85.15.5576 PMC281801

[4] B. Coste, J. Mathur, M. Schmidt, T. J. Earley, S. Ranade, M. J. Petrus, A. E. Dubin, A. Patapoutian, Piezo1 and Piezo2 are essential components of distinct mechanically activated cation channels. Science 330, 55–60 (2010). 20813920 10.1126/science.1193270 PMC3062430

[5] S. Dupont, L. Morsut, M. Aragona, E. Enzo, S. Giulitti, M. Cordenonsi, F. Zanconato, J. Le Digabel, M. Forcato, S. Bicciato, N. Elvassore, S. Piccolo, Role of YAP/TAZ in mechanotransduction. Nature 474, 179–183 (2011). 21654799 10.1038/nature10137

[6] K.-I. Wada, K. Itoga, T. Okano, S. Yonemura, H. Sasaki, Hippo pathway regulation by cell morphology and stress fibers. Development 138, 3907–3914 (2011). 21831922 10.1242/dev.070987

[7] W.-C. Hung, J. R. Yang, C. L. Yankaskas, B. S. Wong, P.-H. Wu, C. Pardo-Pastor, S. A. Serra, M.-J. Chiang, Z. Gu, D. Wirtz, M. A. Valverde, J. T. Yang, J. Zhang, K. Konstantopoulos, Confinement sensing and signal optimization via Piezo1/PKA and myosin II pathways. Cell Rep. 15, 1430–1441 (2016). 27160899 10.1016/j.celrep.2016.04.035 PMC5341576

[8] J. Carrillo-Garcia, V. Herrera-Fernández, S. A. Serra, F. Rubio-Moscardo, M. Vogel-Gonzalez, P. Doñate-Macian, C. F. Hevia, C. Pujades, M. A. Valverde, The mechanosensitive Piezo1 channel controls endosome trafficking for an efficient cytokinetic abscission. Sci. Adv. 7, eabi7785 (2021). 34714681 10.1126/sciadv.abi7785 PMC8555900

[9] A. Lai, P. Thurgood, C. D. Cox, C. Chheang, K. Peter, A. Jaworowski, K. Khoshmanesh, S. Baratchi, Piezo1 response to shear stress is controlled by the components of the extracellular matrix. ACS Appl. Mater. Interfaces 14, 40559–40568 (2022). 36047858 10.1021/acsami.2c09169

[10] J. Li, B. Hou, S. Tumova, K. Muraki, A. Bruns, M. J. Ludlow, A. Sedo, A. J. Hyman, L. McKeown, R. S. Young, N. Y. Yuldasheva, Y. Majeed, L. A. Wilson, B. Rode, M. A. Bailey, H. R. Kim, Z. Fu, D. A. L. Carter, J. Bilton, H. Imrie, P. Ajuh, T. N. Dear, R. M. Cubbon, M. T. Kearney, K. R. Prasad, P. C. Evans, J. F. X. Ainscough, D. J. Beech, Piezo1 integration of vascular architecture with physiological force. Nature 515, 279–282 (2014). 25119035 10.1038/nature13701 PMC4230887

[11] C. Pardo-Pastor, F. Rubio-Moscardo, M. Vogel-González, S. A. Serra, A. Afthinos, S. Mrkonjic, O. Destaing, J. F. Abenza, J. M. Fernández-Fernández, X. Trepat, C. Albiges-Rizo, K. Konstantopoulos, M. A. Valverde, Piezo2 channel regulates RhoA and actin cytoskeleton to promote cell mechanobiological responses. Proc. Natl. Acad. Sci. U.S.A. 115, 1925–1930 (2018). 29432180 10.1073/pnas.1718177115 PMC5828612

[12] M. M. Pathak, J. L. Nourse, T. Tran, J. Hwe, J. Arulmoli, D. T. T. Le, E. Bernardis, L. A. Flanagan, F. Tombola, Stretch-activated ion channel Piezo1 directs lineage choice in human neural stem cells. Proc. Natl. Acad. Sci. U.S.A. 111, 16148–16153 (2014). 25349416 10.1073/pnas.1409802111 PMC4234578

[13] J. R. Holt, W.-Z. Zeng, E. L. Evans, S.-H. Woo, S. Ma, H. Abuwarda, M. Loud, A. Patapoutian, M. M. Pathak, Spatiotemporal dynamics of PIEZO1 localization controls keratinocyte migration during wound healing. eLife 10, e65415 (2021). 34569935 10.7554/eLife.65415 PMC8577841

[14] X. Chen, S. Wanggou, A. Bodalia, M. Zhu, W. Dong, J. J. Fan, W. C. Yin, H.-K. Min, M. Hu, D. Draghici, W. Dou, F. Li, F. J. Coutinho, H. Whetstone, M. M. Kushida, P. B. Dirks, Y. Song, C. Hui, Y. Sun, L.-Y. Wang, X. Li, X. Huang, A feedforward mechanism mediated by mechanosensitive ion channel PIEZO1 and tissue mechanics promotes glioma aggression. Neuron 100, 799–815.e7 (2018). 30344046 10.1016/j.neuron.2018.09.046

[15] G. T. Eisenhoffer, P. D. Loftus, M. Yoshigi, H. Otsuna, C.-B. Chien, P. A. Morcos, J. Rosenblatt, Crowding induces live cell extrusion to maintain homeostatic cell numbers in epithelia. Nature 484, 546–549 (2012). 22504183 10.1038/nature10999 PMC4593481

[16] S. A. Gudipaty, J. Lindblom, P. D. Loftus, M. J. Redd, K. Edes, C. F. Davey, V. Krishnegowda, J. Rosenblatt, Mechanical stretch triggers rapid epithelial cell division through Piezo1. Nature 543, 118–121 (2017). 28199303 10.1038/nature21407 PMC5334365

[17] B. W. Benham-Pyle, B. L. Pruitt, W. J. Nelson, Mechanical strain induces E-cadherin-dependent Yap1 and β-catenin activation to drive cell cycle entry. Science 348, 1024–1027 (2015). 26023140 10.1126/science.aaa4559 PMC4572847

[18] Y. Han, C. Liu, D. Zhang, H. Men, L. Huo, Q. Geng, S. Wang, Y. Gao, W. Zhang, Y. Zhang, Z. Jia, Mechanosensitive ion channel Piezo1 promotes prostate cancer development through the activation of the Akt/mTOR pathway and acceleration of cell cycle. Int. J. Oncol. 55, 629–644 (2019). 31322184 10.3892/ijo.2019.4839 PMC6685593

[19] R. Avraham, Y. Yarden, Feedback regulation of EGFR signalling: Decision making by early and delayed loops. Nat. Rev. Mol. Cell Biol. 12, 104–117 (2011). 21252999 10.1038/nrm3048

[20] P. E. Farahani, S. B. Lemke, E. Dine, G. Uribe, J. E. Toettcher, C. M. Nelson, Substratum stiffness regulates Erk signaling dynamics through receptor-level control. Cell Rep. 37, 110181 (2021). 34965432 10.1016/j.celrep.2021.110181 PMC8756379

[21] M. A. Lemmon, J. Schlessinger, Cell signaling by receptor tyrosine kinases. Cell 141, 1117–1134 (2010). 20602996 10.1016/j.cell.2010.06.011 PMC2914105

[22] N. Prenzel, E. Zwick, H. Daub, M. Leserer, R. Abraham, C. Wallasch, A. Ullrich, EGF receptor transactivation by G-protein-coupled receptors requires metalloproteinase cleavage of proHB-EGF. Nature 402, 884–888 (1999). 10622253 10.1038/47260

[23] L. B. Rosen, M. E. Greenberg, Stimulation of growth factor receptor signal transduction by activation of voltage-sensitive calcium channels. Proc. Natl. Acad. Sci. U.S.A. 93, 1113–1118 (1996). 8577724 10.1073/pnas.93.3.1113 PMC40040

[24] S. Sigismund, E. Argenzio, D. Tosoni, E. Cavallaro, S. Polo, P. P. Di Fiore, Clathrin-mediated internalization is essential for sustained EGFR signaling but dispensable for degradation. Dev. Cell 15, 209–219 (2008). 18694561 10.1016/j.devcel.2008.06.012

[25] N. Hino, L. Rossetti, A. Marín-Llauradó, K. Aoki, X. Trepat, M. Matsuda, T. Hirashima, ERK-mediated mechanochemical waves direct collective cell polarization. Dev. Cell 53, 646–660.e8 (2020). 32497487 10.1016/j.devcel.2020.05.011

[26] H. Iwasaki, S. Eguchi, H. Ueno, F. Marumo, Y. Hirata, Mechanical stretch stimulates growth of vascular smooth muscle cells via epidermal growth factor receptor. Am. J. Physiol. Heart Circ. Physiol. 278, H521–H529 (2000). 10666084 10.1152/ajpheart.2000.278.2.H521

[27] J.-H. Kim, A. R. Asthagiri, Matrix stiffening sensitizes epithelial cells to EGF and enables the loss of contact inhibition of proliferation. J. Cell Sci. 124, 1280–1287 (2011). 21429934 10.1242/jcs.078394 PMC3065384

[28] S. K. Kuwada, X. Li, Integrin α5/β1 mediates fibronectin-dependent epithelial cell proliferation through epidermal growth factor receptor activation. Mol. Biol. Cell 11, 2485–2496 (2000). 10888683 10.1091/mbc.11.7.2485 PMC14934

[29] L. Moro, M. Venturino, C. Bozzo, L. Silengo, F. Altruda, L. Beguinot, G. Tarone, P. Defilippi, Integrins induce activation of EGF receptor: Role in MAP kinase induction and adhesion-dependent cell survival. EMBO J. 17, 6622–6632 (1998). 9822606 10.1093/emboj/17.22.6622 PMC1171008

[30] L. O. Murphy, J. Blenis, MAPK signal specificity: The right place at the right time. Trends Biochem. Sci. 31, 268–275 (2006). 16603362 10.1016/j.tibs.2006.03.009

[31] V. Umesh, A. D. Rape, T. A. Ulrich, S. Kumar, Microenvironmental stiffness enhances glioma cell proliferation by stimulating epidermal growth factor receptor signaling. PLOS ONE 9, e101771 (2014). 25000176 10.1371/journal.pone.0101771 PMC4084995

[32] S. Yano, M. Komine, M. Fujimoto, H. Okochi, K. Tamaki, Mechanical stretching in vitro regulates signal transduction pathways and cellular proliferation in human epidermal keratinocytes. J. Invest. Dermatol. 122, 783–790 (2004). 15086566 10.1111/j.0022-202X.2004.22328.x

[33] Z. Wang, M. F. Moran, Requirement for the adapter protein GRB2 in EGF receptor endocytosis. Science 272, 1935–1938 (1996). 8658166 10.1126/science.272.5270.1935

[34] M. V. Grandal, L. M. Grøvdal, L. Henriksen, M. H. Andersen, M. R. Holst, I. H. Madshus, B. van Deurs, Differential roles of Grb2 and AP-2 in p38MAPK- and EGF-induced EGFR internalization. Traffic 13, 576–585 (2012). 22192528 10.1111/j.1600-0854.2011.01322.x

[35] S. Sigismund, V. Algisi, G. Nappo, A. Conte, R. Pascolutti, A. Cuomo, T. Bonaldi, E. Argenzio, L. G. G. C. Verhoef, E. Maspero, F. Bianchi, F. Capuani, A. Ciliberto, S. Polo, P. P. Di Fiore, Threshold-controlled ubiquitination of the EGFR directs receptor fate. EMBO J. 32, 2140–2157 (2013). 23799367 10.1038/emboj.2013.149 PMC3730230

[36] S. Sigismund, T. Woelk, C. Puri, E. Maspero, C. Tacchetti, P. Transidico, P. P. Di Fiore, S. Polo, Clathrin-independent endocytosis of ubiquitinated cargos. Proc. Natl. Acad. Sci. U.S.A. 102, 2760–2765 (2005). 15701692 10.1073/pnas.0409817102 PMC549482

[37] D. J. Tschumperlin, G. Dai, I. V. Maly, T. Kikuchi, L. H. Laiho, A. K. McVittie, K. J. Haley, C. M. Lilly, P. T. C. So, D. A. Lauffenburger, R. D. Kamm, J. M. Drazen, Mechanotransduction through growth-factor shedding into the extracellular space. Nature 429, 83–86 (2004). 15103386 10.1038/nature02543 PMC5539413

[38] L. Moro, L. Dolce, S. Cabodi, E. Bergatto, E. Boeri Erba, M. Smeriglio, E. Turco, S. F. Retta, M. G. Giuffrida, M. Venturino, J. Godovac-Zimmermann, A. Conti, E. Schaefer, L. Beguinot, C. Tacchetti, P. Gaggini, L. Silengo, G. Tarone, P. Defilippi, Integrin-induced epidermal growth factor (EGF) receptor activation requires c-Src and p130Cas and leads to phosphorylation of specific EGF receptor tyrosines. J. Biol. Chem. 277, 9405–9414 (2002). 11756413 10.1074/jbc.M109101200

[39] M. Saxena, S. Liu, B. Yang, C. Hajal, R. Changede, J. Hu, H. Wolfenson, J. Hone, M. P. Sheetz, EGFR and HER2 activate rigidity sensing only on rigid matrices. Nat. Mater. 16, 775–781 (2017). 28459445 10.1038/nmat4893 PMC5920513

[40] J. Li, C. Ma, Y. Huang, J. Luo, C. Huang, Differential requirement of EGF receptor and its tyrosine kinase for AP-1 transactivation induced by EGF and TPA. Oncogene 22, 211–219 (2003). 12527890 10.1038/sj.onc.1206102

[41] M. P. Oksvold, E. Skarpen, B. Lindeman, N. Roos, H. S. Huitfeldt, Immunocytochemical localization of Shc and activated EGF receptor in early endosomes after EGF stimulation of HeLa cells. J. Histochem. Cytochem. 48, 21–33 (2000). 10653583 10.1177/002215540004800103

[42] M. Perez Verdaguer, T. Zhang, J. A. Paulo, S. Gygi, S. C. Watkins, H. Sakurai, A. Sorkin, Mechanism of p38 MAPK–induced EGFR endocytosis and its crosstalk with ligand-induced pathways. J. Cell Biol. 220, e202102005 (2021). 34032851 10.1083/jcb.202102005 PMC8155814

[43] B. J. McHugh, R. Buttery, Y. Lad, S. Banks, C. Haslett, T. Sethi, Integrin activation by Fam38A uses a novel mechanism of R-Ras targeting to the endoplasmic reticulum. J. Cell Sci. 123, 51–61 (2010). 20016066 10.1242/jcs.056424 PMC2794710

[44] R. Syeda, J. Xu, A. E. Dubin, B. Coste, J. Mathur, T. Huynh, J. Matzen, J. Lao, D. C. Tully, I. H. Engels, H. M. Petrassi, A. M. Schumacher, M. Montal, M. Bandell, A. Patapoutian, Chemical activation of the mechanotransduction channel Piezo1. eLife 4, e07369 (2015). 26001275 10.7554/eLife.07369 PMC4456433

[45] M. R. Frey, R. S. Dise, K. L. Edelblum, D. B. Polk, p38 kinase regulates epidermal growth factor receptor downregulation and cellular migration. EMBO J. 25, 5683–5692 (2006). 17139251 10.1038/sj.emboj.7601457 PMC1698902

[46] T. Tanaka, Y. Zhou, T. Ozawa, R. Okizono, A. Banba, T. Yamamura, E. Oga, A. Muraguchi, H. Sakurai, Ligand-activated epidermal growth factor receptor (EGFR) signaling governs endocytic trafficking of unliganded receptor monomers by non-canonical phosphorylation. J. Biol. Chem. 293, 2288–2301 (2018). 29255092 10.1074/jbc.M117.811299 PMC5818182

[47] Y. Wei, Z. Zou, N. Becker, M. Anderson, R. Sumpter, G. Xiao, L. Kinch, P. Koduru, C. S. Christudass, R. W. Veltri, N. V. Grishin, M. Peyton, J. Minna, G. Bhagat, B. Levine, EGFR-mediated beclin 1 phosphorylation in autophagy suppression, tumor progression, and tumor chemoresistance. Cell 154, 1269–1284 (2013). 24034250 10.1016/j.cell.2013.08.015 PMC3917713

[48] S. Vergarajauregui, A. S. Miguel, R. Puertollano, Activation of p38 mitogen-activated protein kinase promotes epidermal growth factor receptor internalization. Traffic 7, 686–698 (2006). 16683917 10.1111/j.1600-0854.2006.00420.x PMC1479226

[49] Y. Zwang, Y. Yarden, p38 MAP kinase mediates stress-induced internalization of EGFR: Implications for cancer chemotherapy. EMBO J. 25, 4195–4206 (2006). 16932740 10.1038/sj.emboj.7601297 PMC1570432

[50] S. Adachi, H. Natsume, J. Yamauchi, R. Matsushima-Nishiwaki, A. K. Joe, H. Moriwaki, O. Kozawa, p38 MAP kinase controls EGF receptor downregulation via phosphorylation at Ser1046/1047. Cancer Lett. 277, 108–113 (2009). 19138820 10.1016/j.canlet.2008.11.034

[51] N. M. Blythe, K. Muraki, M. J. Ludlow, V. Stylianidis, H. T. J. Gilbert, E. L. Evans, K. Cuthbertson, R. Foster, J. Swift, J. Li, M. J. Drinkhill, F. A. van Nieuwenhoven, K. E. Porter, D. J. Beech, N. A. Turner, Mechanically activated Piezo1 channels of cardiac fibroblasts stimulate p38 mitogen-activated protein kinase activity and interleukin-6 secretion. J. Biol. Chem. 294, 17395–17408 (2019). 31586031 10.1074/jbc.RA119.009167 PMC6873183

[52] P. Wu, P. Wee, J. Jiang, X. Chen, Z. Wang, Differential regulation of transcription factors by location-specific EGF receptor signaling via a spatio-temporal interplay of ERK activation. PLOS ONE 7, e41354 (2012). 22984397 10.1371/journal.pone.0041354 PMC3440385

[53] T. Panciera, L. Azzolin, A. Fujimura, D. Di Biagio, C. Frasson, S. Bresolin, S. Soligo, G. Basso, S. Bicciato, A. Rosato, M. Cordenonsi, S. Piccolo, Induction of expandable tissue-specific stem/progenitor cells through transient expression of YAP/TAZ. Cell Stem Cell 19, 725–737 (2016). 27641305 10.1016/j.stem.2016.08.009 PMC5145813

[54] S. Yui, L. Azzolin, M. Maimets, M. T. Pedersen, R. P. Fordham, S. L. Hansen, H. L. Larsen, J. Guiu, M. R. P. Alves, C. F. Rundsten, J. V. Johansen, Y. Li, C. D. Madsen, T. Nakamura, M. Watanabe, O. H. Nielsen, P. J. Schweiger, S. Piccolo, K. B. Jensen, YAP/TAZ-dependent reprogramming of colonic epithelium links ECM remodeling to tissue regeneration. Cell Stem Cell 22, 35–49.e7 (2018). 29249464 10.1016/j.stem.2017.11.001 PMC5766831

[55] F. Zanconato, M. Forcato, G. Battilana, L. Azzolin, E. Quaranta, B. Bodega, A. Rosato, S. Bicciato, M. Cordenonsi, S. Piccolo, Genome-wide association between YAP/TAZ/TEAD and AP-1 at enhancers drives oncogenic growth. Nat. Cell Biol. 17, 1218–1227 (2015). 26258633 10.1038/ncb3216 PMC6186417

[56] A. Franck, J. Lainé, G. Moulay, M. Trichet, C. Gentil, A. Fongy, A. Bigot, S. Benkhelifa-Ziyyat, E. Lacène, M. Thao Bui, G. Brochier, P. Guicheney, S. Sacconi, V. Mouly, N. Romero, C. Coirault, M. Bitoun, S. Vassilopoulos, Mechanosensitive clathrin platforms anchor desmin intermediate filaments in skeletal muscle, (preprint, Cell Biology, 2018).

[57] Y. Hart, U. Alon, The utility of paradoxical components in biological circuits. Mol. Cell 49, 213–221 (2013). 23352242 10.1016/j.molcel.2013.01.004

[58] Y. Brüggemann, L. S. Karajannis, A. Stanoev, W. Stallaert, P. I. H. Bastiaens, Growth factor–dependent ErbB vesicular dynamics couple receptor signaling to spatially and functionally distinct Erk pools. Sci. Signal. 14, eabd9943 (2021). 34006609 10.1126/scisignal.abd9943

[59] W. Stallaert, Y. Brüggemann, O. Sabet, L. Baak, M. Gattiglio, P. I. H. Bastiaens, Contact inhibitory Eph signaling suppresses EGF-promoted cell migration by decoupling EGFR activity from vesicular recycling. Sci. Signal. 11, eaat0114 (2018). 30065026 10.1126/scisignal.aat0114

[60] B. P. Ceresa, Spatial regulation of epidermal growth factor receptor signaling by endocytosis. Int. J. Mol. Sci. 14, 72–87 (2013). 10.3390/ijms14010072 PMC3565252 23344022

[61] E. Barbieri, P. P. Di Fiore, S. Sigismund, Endocytic control of signaling at the plasma membrane. Curr. Opin. Cell Biol. 39, 21–27 (2016). 26872272 10.1016/j.ceb.2016.01.012

[62] A. Tomas, S. O. Vaughan, T. Burgoyne, A. Sorkin, J. A. Hartley, D. Hochhauser, C. E. Futter, WASH and Tsg101/ALIX-dependent diversion of stress-internalized EGFR from the canonical endocytic pathway. Nat. Commun. 6, 7324 (2015). 26066081 10.1038/ncomms8324 PMC4490399

[63] S.-A. Nikou, C. Zhou, J. S. Griffiths, N. K. Kotowicz, B. M. Coleman, M. J. Green, D. L. Moyes, S. L. Gaffen, J. R. Naglik, P. J. Parker, The *Candida albicans* toxin candidalysin mediates distinct epithelial inflammatory responses through p38 and EGFR-ERK pathways. Sci. Signal. 15, eabj6915 (2022). 35380879 10.1126/scisignal.abj6915 PMC7612652

[64] M.-J. Kim, J.-Y. Byun, C.-H. Yun, I.-C. Park, K.-H. Lee, S.-J. Lee, c-Src-p38 mitogen-activated protein kinase signaling is required for Akt activation in response to ionizing radiation. Mol. Cancer Res. 6, 1872–1880 (2008). 19074832 10.1158/1541-7786.MCR-08-0084

[65] I. Canobbio, L. Cipolla, G. F. Guidetti, D. Manganaro, C. Visconte, S. Kim, M. Okigaki, M. Falasca, S. P. Kunapuli, M. Torti, The focal adhesion kinase Pyk2 links Ca2^+^ signalling to Src family kinase activation and protein tyrosine phosphorylation in thrombin-stimulated platelets. Biochem. J. 469, 199–210 (2015). 25967238 10.1042/BJ20150048

[66] A. N. Kim, W.-K. Jeon, K.-H. Lim, H.-Y. Lee, W. J. Kim, B.-C. Kim, Fyn mediates transforming growth factor-beta1-induced down-regulation of E-cadherin in human A549 lung cancer cells. Biochem. Biophys. Res. Commun. 407, 181–184 (2011). 21371426 10.1016/j.bbrc.2011.02.134

[67] L. A. Samayawardhena, J. Hu, P. L. Stein, A. W. B. Craig, Fyn kinase acts upstream of Shp2 and p38 mitogen-activated protein kinase to promote chemotaxis of mast cells towards stem cell factor. Cell. Signal. 18, 1447–1454 (2006). 16442778 10.1016/j.cellsig.2005.11.005

[68] P. Sánchez-González, K. Jellali, A. Villalobo, Calmodulin-mediated regulation of the epidermal growth factor receptor. FEBS J. 277, 327–342 (2010). 19951361 10.1111/j.1742-4658.2009.07469.x

[69] A. D. Schwartz, C. L. Hall, L. E. Barney, C. C. Babbitt, S. R. Peyton, Integrin α6 and EGFR signaling converge at mechanosensitive calpain 2. Biomaterials 178, 73–82 (2018). 29909039 10.1016/j.biomaterials.2018.05.056 PMC6211197

[70] M. E. Fernández-Sánchez, S. Barbier, J. Whitehead, G. Béalle, A. Michel, H. Latorre-Ossa, C. Rey, L. Fouassier, A. Claperon, L. Brullé, E. Girard, N. Servant, T. Rio-Frio, H. Marie, S. Lesieur, C. Housset, J.-L. Gennisson, M. Tanter, C. Ménager, S. Fre, S. Robine, E. Farge, Mechanical induction of the tumorigenic β-catenin pathway by tumour growth pressure. Nature 523, 92–95 (2015). 25970250 10.1038/nature14329

[71] M. J. Paszek, N. Zahir, K. R. Johnson, J. N. Lakins, G. I. Rozenberg, A. Gefen, C. A. Reinhart-King, S. S. Margulies, M. Dembo, D. Boettiger, D. A. Hammer, V. M. Weaver, Tensional homeostasis and the malignant phenotype. Cancer Cell 8, 241–254 (2005). 16169468 10.1016/j.ccr.2005.08.010

[72] R. Thomas, Z. Weihua, Rethink of EGFR in cancer with its kinase independent function on board. Front. Oncol. 9, 800 (2019). 31508364 10.3389/fonc.2019.00800 PMC6716122

[73] X. Tan, N. Thapa, Y. Sun, R. A. Anderson, A kinase-independent role for EGF receptor in autophagy initiation. Cell 160, 145–160 (2015). 25594178 10.1016/j.cell.2014.12.006 PMC4297316

[74] F. Broders-Bondon, T. H. N. Ho-Bouldoires, M.-E. Fernandez-Sanchez, E. Farge, Mechanotransduction in tumor progression: The dark side of the force. J. Cell Biol. 217, 1571–1587 (2018). 29467174 10.1083/jcb.201701039 PMC5940296

[75] T. Zulueta-Coarasa, J. Fadul, M. Ahmed, J. Rosenblatt, Physical confinement promotes mesenchymal trans-differentiation of invading transformed cells in vivo. iScience 25, 105330 (2022). 36325066 10.1016/j.isci.2022.105330 PMC9618776

[76] Y. R. Guo, R. MacKinnon, Structure-based membrane dome mechanism for Piezo mechanosensitivity. eLife 6, e33660 (2017). 29231809 10.7554/eLife.33660 PMC5788504

[77] F. Baschieri, S. Dayot, N. Elkhatib, N. Ly, A. Capmany, K. Schauer, T. Betz, D. M. Vignjevic, R. Poincloux, G. Montagnac, Frustrated endocytosis controls contractility-independent mechanotransduction at clathrin-coated structures. Nat. Commun. 9, 3825 (2018). 30237420 10.1038/s41467-018-06367-y PMC6148028

[78] S. Boulant, C. Kural, J.-C. Zeeh, F. Ubelmann, T. Kirchhausen, Actin dynamics counteract membrane tension during clathrin-mediated endocytosis. Nat. Cell Biol. 13, 1124–1131 (2011). 21841790 10.1038/ncb2307 PMC3167020

[79] J. Geng, Y. Shi, J. Zhang, B. Yang, P. Wang, W. Yuan, H. Zhao, J. Li, F. Qin, L. Hong, C. Xie, X. Deng, Y. Sun, C. Wu, L. Chen, D. Zhou, TLR4 signalling via Piezo1 engages and enhances the macrophage mediated host response during bacterial infection. Nat. Commun. 12, 3519 (2021). 34112781 10.1038/s41467-021-23683-y PMC8192512

[80] C. D. Cox, C. Bae, L. Ziegler, S. Hartley, V. Nikolova-Krstevski, P. R. Rohde, C.-A. Ng, F. Sachs, P. A. Gottlieb, B. Martinac, Removal of the mechanoprotective influence of the cytoskeleton reveals PIEZO1 is gated by bilayer tension. Nat. Commun. 7, 10366 (2016). 26785635 10.1038/ncomms10366 PMC4735864

[81] R. Syeda, M. N. Florendo, C. D. Cox, J. M. Kefauver, J. S. Santos, B. Martinac, A. Patapoutian, Piezo1 channels are inherently mechanosensitive. Cell Rep. 17, 1739–1746 (2016). 27829145 10.1016/j.celrep.2016.10.033 PMC5129625

[82] H. De Belly, E. K. Paluch, K. J. Chalut, Interplay between mechanics and signalling in regulating cell fate. Nat. Rev. Mol. Cell Biol. 23, 465–480 (2022). 35365816 10.1038/s41580-022-00472-z

[83] J. G. Joseph, A. P. Liu, Mechanical regulation of endocytosis: New insights and recent advances. Adv. Biosyst. 4, 1900278 (2020). 10.1002/adbi.202000155 33179871

[84] J. H. Li, V. Trivedi, A. Diz-Muñoz, Understanding the interplay of membrane trafficking, cell surface mechanics, and stem cell differentiation. Semin. Cell Dev. Biol. 133, 123–134 (2023). 35641408 10.1016/j.semcdb.2022.05.010 PMC9703995

[85] F. D. Camargo, S. Gokhale, J. B. Johnnidis, D. Fu, G. W. Bell, R. Jaenisch, T. R. Brummelkamp, YAP1 increases organ size and expands undifferentiated progenitor cells. Curr. Biol. 17, 2054–2060 (2007). 17980593 10.1016/j.cub.2007.10.039

[86] D. M. Freed, N. J. Bessman, A. Kiyatkin, E. Salazar-Cavazos, P. O. Byrne, J. O. Moore, C. C. Valley, K. M. Ferguson, D. J. Leahy, D. S. Lidke, M. A. Lemmon, EGFR ligands differentially stabilize receptor dimers to specify signaling kinetics. Cell 171, 683–695.e18 (2017). 28988771 10.1016/j.cell.2017.09.017 PMC5650921

[87] C. Poon, Measuring the density and viscosity of culture media for optimized computational fluid dynamics analysis of *in vitro* devices, (preprint, Bioengineering, 2020); 10.1101/2020.08.25.266221. 34911025

[88] J. Schindelin, I. Arganda-Carreras, E. Frise, V. Kaynig, M. Longair, T. Pietzsch, S. Preibisch, C. Rueden, S. Saalfeld, B. Schmid, J.-Y. Tinevez, D. J. White, V. Hartenstein, K. Eliceiri, P. Tomancak, A. Cardona, Fiji: An open-source platform for biological-image analysis. Nat. Methods 9, 676–682 (2012). 22743772 10.1038/nmeth.2019 PMC3855844

[89] K. M. Akram, L. L. Yates, R. Mongey, S. Rothery, D. C. A. Gaboriau, J. Sanderson, M. Hind, M. Griffiths, C. H. Dean, Live imaging of alveologenesis in precision-cut lung slices reveals dynamic epithelial cell behaviour. Nat. Commun. 10, 1178 (2019). 30862802 10.1038/s41467-019-09067-3 PMC6414680

[90] C. McQuin, A. Goodman, V. Chernyshev, L. Kamentsky, B. A. Cimini, K. W. Karhohs, M. Doan, L. Ding, S. M. Rafelski, D. Thirstrup, W. Wiegraebe, S. Singh, T. Becker, J. C. Caicedo, A. E. Carpenter, CellProfiler 3.0: Next-generation image processing for biology. PLoS Biol. 16, e2005970 (2018). 29969450 10.1371/journal.pbio.2005970 PMC6029841

[91] K. W. Dunn, M. M. Kamocka, J. H. McDonald, A practical guide to evaluating colocalization in biological microscopy. Am. J. Physiol. Cell Physiol. 300, C723–C742 (2011). 21209361 10.1152/ajpcell.00462.2010 PMC3074624

