# Supplementary material for: Piezo1 activates noncanonical EGFR endocytosis and signaling
Source: Sci Adv. 2023 Sep 27;9(39):eadi1328. doi: 10.1126/sciadv.adi1328 (PMC10530101; doi:10.1126/sciadv.adi1328)
Supplement: Supplementary file 1 — Figs. S1 to S3 Legend for table S1 Legend for example cell profiler pipeline for EGFR puncta detection [file sciadv.adi1328_sm.pdf]

Science Advances
AAAS

Supplementary Materials for

Piezo1 activates noncanonical EGFR endocytosis and signaling

Carlos Pardo-Pastor and Jody Rosenblatt

Corresponding author: Carlos Pardo-Pastor carlos.pardo-pastor@kcl.ac.uk, carlos.pardo.pastor@gmail.com

Sci. Adv. **9**, eadi1328 (2023)
DOI: 10.1126/sciadv.adi1328

The PDF file includes:

Figs. S1 to S3
Legend for table S1
Legend for example cell profiler pipeline for EGFR puncta detection

Other Supplementary Material for this manuscript includes the following:

Table S1
Example cell profiler pipeline for EGFR puncta detection

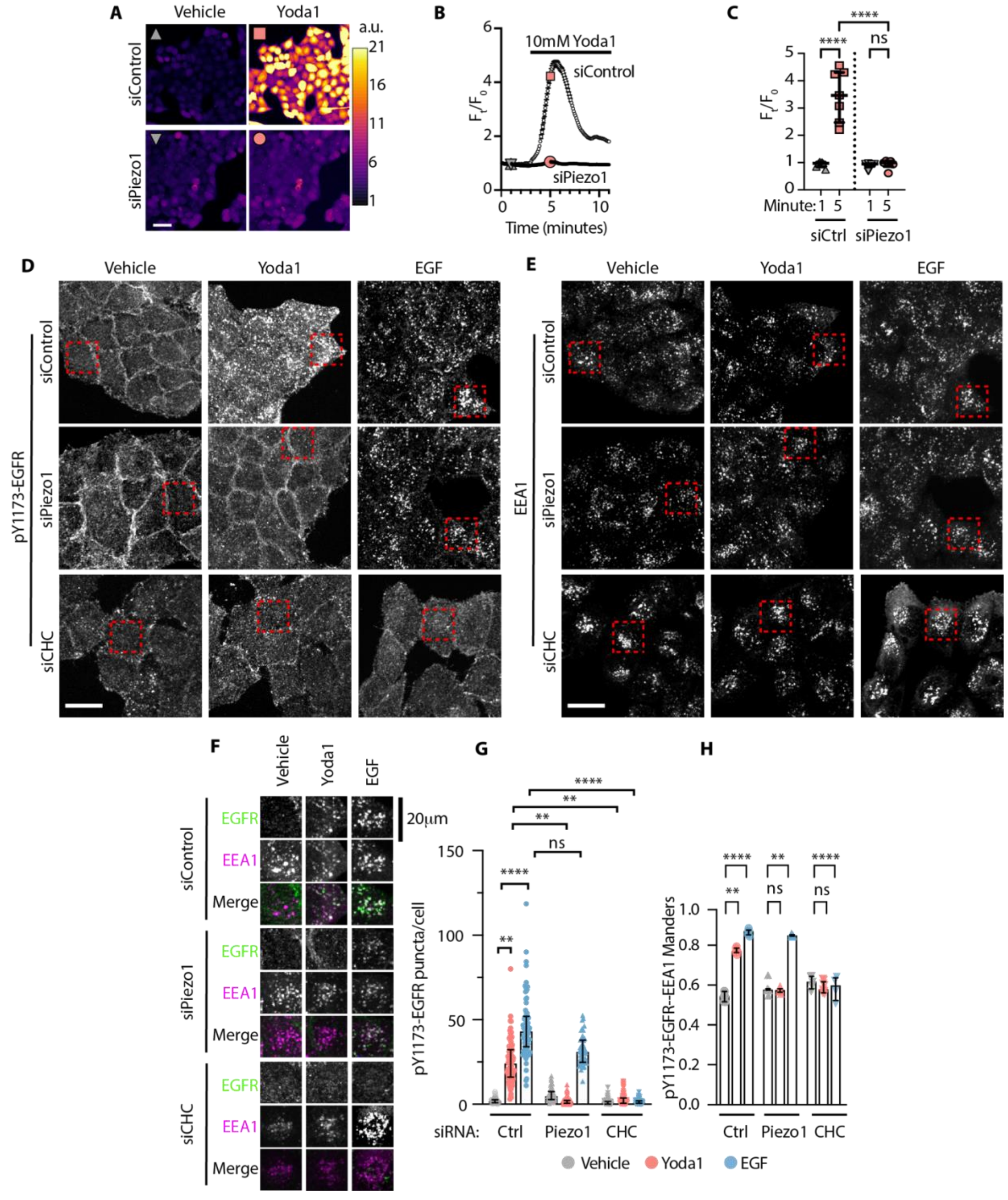
A
Vehicle
Yoda1
a.u.
21
16
11
6
1
siControl
siPiezo1
B
10mM Yoda1
siControl
siPiezo1
Time (minutes)
C
Minute: 1 5 1 5
siCtrl siPiezo1
ns
D
Vehicle
Yoda1
EGF
pY1173-EGFR
siControl
siPiezo1
siCHC
E
Vehicle
Yoda1
EGF
EEA1
siControl
siPiezo1
siCHC
F
Vehicle
Yoda1
EGF
20μm
EGFR
EEA1
Merge
siControl
siPiezo1
siCHC
G
pY1173-EGFR puncta/cell
siRNA: Ctrl Piezo1 CHC
ns
H
pY1173-EGFR--EEA1 Manders
Ctrl Piezo1 CHC
ns
Vehicle Yoda1 EGF

Fig. S1. Clathrin mediated EGFR endocytosis in response to Piezo1 activation.

A, B, C: Representative pseudo-coloured micrographs (A), mean traces (B), and summary statistics (C) of fluorescence intensity in siRNA-transfected HeLa cells loaded with the Ca^{2+} indicator Calbryte520AM before and after 10µM Yoda1 treatment. 3 transfections, N=7 (siCtrl), 6 (siPiezo1), comprising >1000 cells. **D, E:** Matched representative maximum intensity projections of pY1173-EGFR (D) or EEA1 (E) stainings in siControl, siPiezo1 or siCHC HeLa cells treated with vehicle, 10µM Yoda1, or 10ng/mL EGF. **F:** Matched magnifications of inserts indicated in red in D and E. **G:** Counts of pY1173-EGFR puncta per cell. Each symbol represents a cell. siCtrl (431 Vehicle, 231 Yoda1, 221 EGF), siPiezo1 (611 Vehicle, 702 Yoda1, 298 EGF), siCHC (187 Vehicle, 371 Yoda1, 236 EGF) 3 experiments from 3 siRNA transfections. **H:** Manders colocalization coefficient between pY1173-EGFR and EEA1. Each symbol represents a picture from ≥4 independent experiments with ≥20 cells per picture. siControl 7 (Vehicle), 7 (Yoda1), 6 (EGF); siPiezo1 8 (Vehicle), 6 (Yoda1), 11 (EGF); siCHC 5 (Vehicle), 9 (Yoda1), 4 (EGF)). Scale bar = 20µm. Error bars = median±interquartile range. ns= non-significant, ** $p<0.01$, **** $p< 0.0001$, one-way ANOVA followed by Kruskal-Wallis post-hoc test with Dunn’s correction for multiple comparisons.

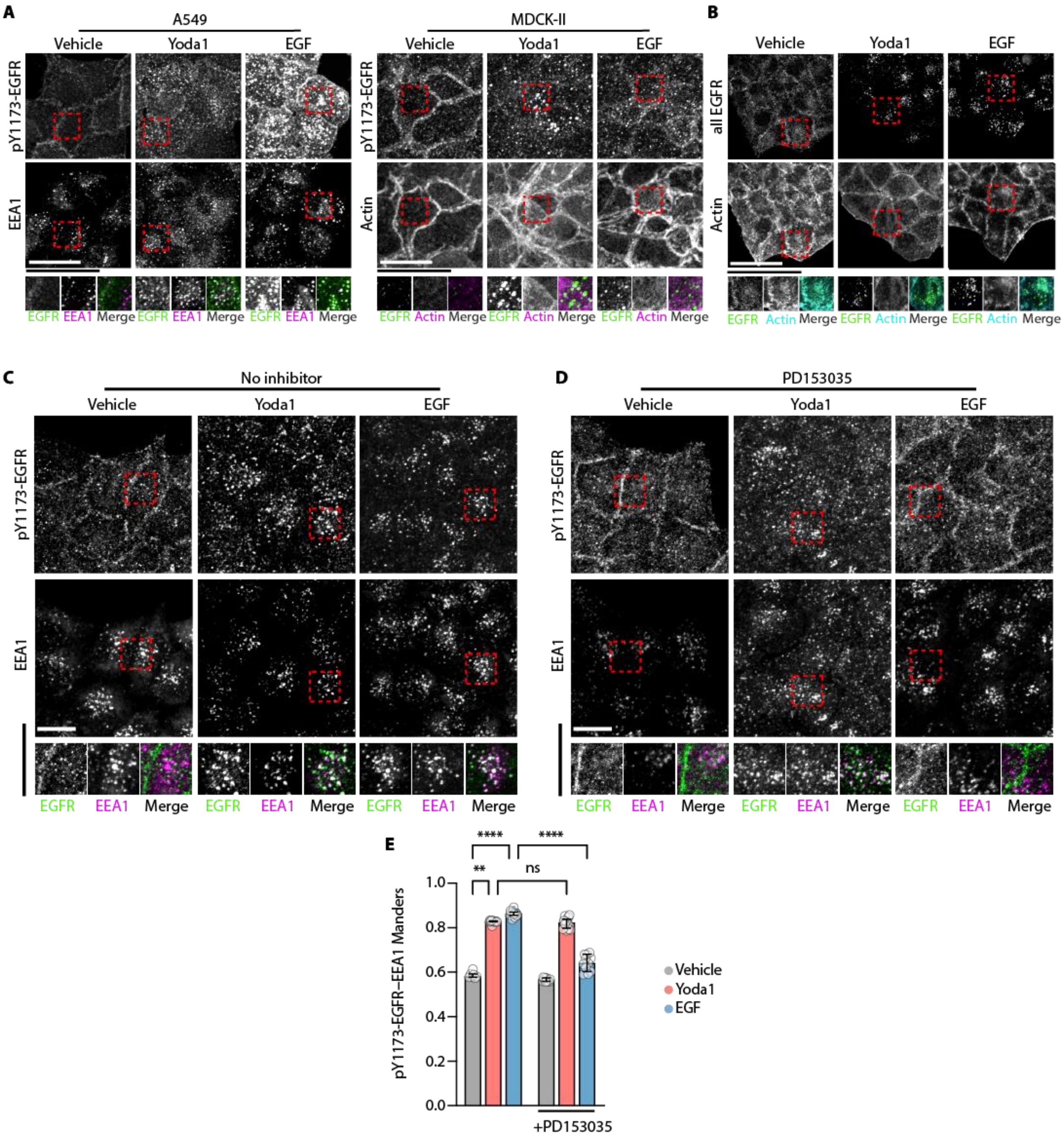

A
A549
Vehicle
Yoda1
EGF
pY1173-EGFR
EEA1
EGFR EEA1 Merge
MDCK-II
Actin
EGFR Actin Merge
B
all EGFR
EGFR Actin Merge
C
No inhibitor
D
PD153035
E
pY1173-EGFR–EEA1 Manders
****
**
ns
1.0
0.8
0.6
0.4
0.2
0.0
+PD153035
Vehicle
Yoda1
EGF

Fig. S2. EGFR CME in response to Piezo1 activation is conserved and independent of EGFR tyrosine kinase activity.

A-D: Representative maximum intensity projections of pY1173-EGFR, EEA1, and Actin stainings in A549 and MDCK-II cells (A, C, D) or of total EGFR and Actin in HeLa cells (B) treated for 15min as indicated. **E:** Manders colocalization coefficient between pY1173-EGFR and EEA1. Each symbol represents a picture from ≥4 independent experiments with ≥20 cells per picture. 7 (Vehicle), 12 (Yoda1), 14 (EGF); 10 (PD15305), 16 (Yoda1+ PD15305), 11 (EGF+ PD15305). Scale bar = 20µm. Error bars = median±interquartile range. ns= non-significant, ** $p<0.01$, **** $p< 0.0001$, one-way ANOVA followed by Kruskal-Wallis post-hoc test with Dunn's correction for multiple comparisons.

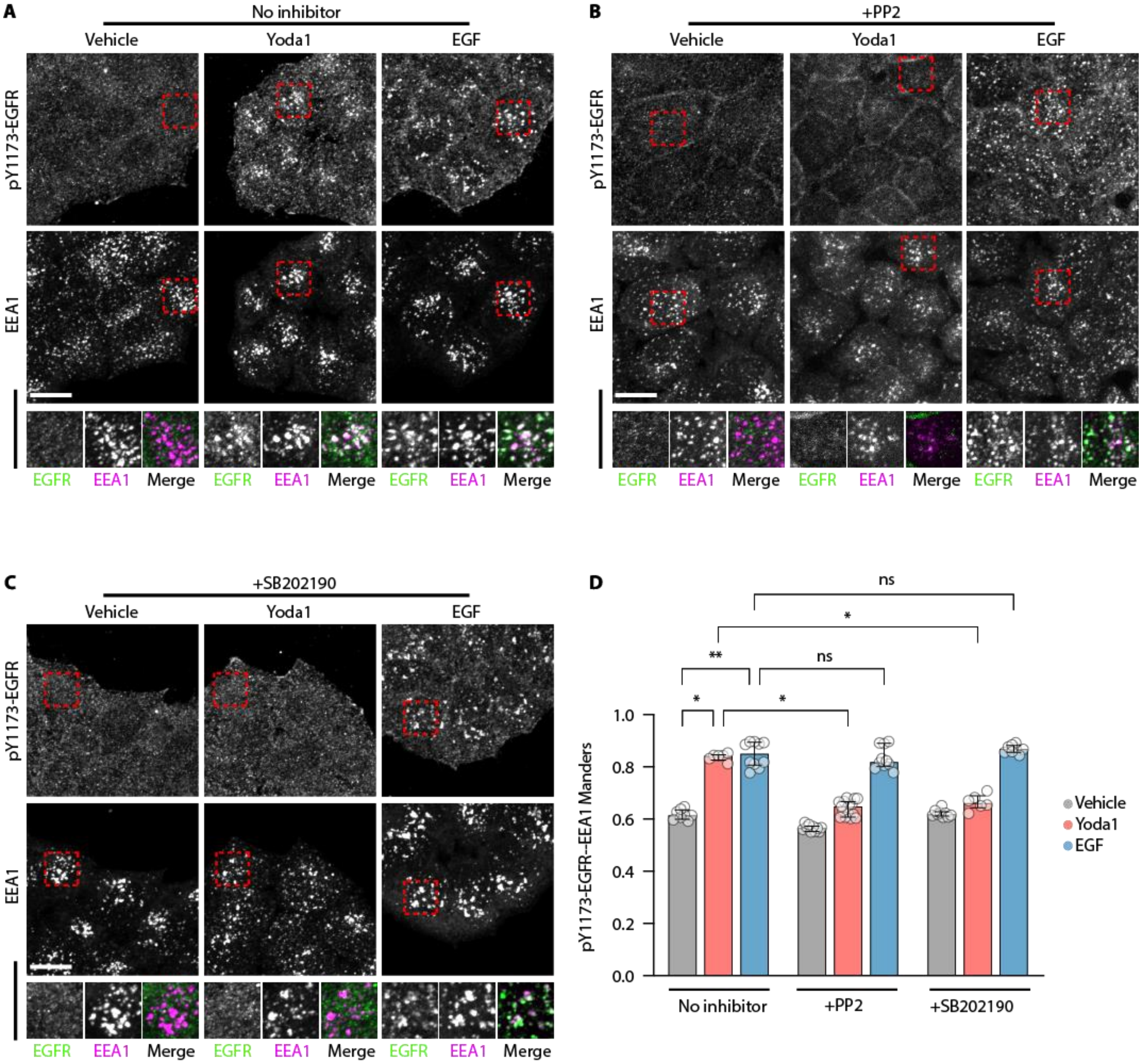

A
No inhibitor
Vehicle
Yoda1
EGF
pY1173-EGFR
EEA1
EGFR
EEA1
Merge
B
+PP2
C
+SB202190
D
pY1173-EGFR–EEA1 Manders
1.0
0.8
0.6
0.4
0.2
0.0
ns
*
**
*
Vehicle
Yoda1
EGF
No inhibitor
+PP2
+SB202190

Fig. S3. EGFR CME in response to Piezo1 activation requires SFK and p38.

A-C: Representative maximum intensity projections of pY1173-EGFR and EEA1. Red discontinuous squares define regions magnified at the bottom of each picture. **D:** Manders colocalization coefficient between pY1173-EGFR and EEA1. Each symbol represents a picture (n) N≥4 independent experiments with ≥20 cells per picture. n= 8 (Vehicle), 6 (Yoda1), 10 (EGF); 10 (PP2), 15 (Yoda1+ PP2), 10 (EGF+ PP2); 9 (SB202190), 6 (Yoda1+ SB202190), 8 (EGF+ SB202190). Scale bar = 20μm. Error bars = median±interquartile range. ns= non-significant, * $p<0.05$, ** $p< 0.01$, one-way ANOVA followed by Kruskal-Wallis post-hoc test with Dunn's correction for multiple comparisons.

Other Supplementary Materials for this manuscript include the following:

- Supplementary table 1 containing values used for graph building and statistical analysis.

- Example Cell Profiler pipeline for EGFR puncta detection.
